# Outcomes of sequential keratoplasty for bilateral gelatinous drop-like corneal dystrophy: A case report

**DOI:** 10.1097/MD.0000000000051066

**Published:** 2026-10-09

**Authors:** Mohammed M. Abusayf, Rana Alhumaemydi, Abeer Alkhodier, Majed AlKharashi

**Affiliations:** a Department of Ophthalmology, College of Medicine, King Saud University, Riyadh, Saudi Arabia; b Refractive Surgery and Myopia Research Group, Ophthalmology and Visual Sciences Research Center, Department of Ophthalmology, King Saud University, Riyadh, Saudi Arabia; c King Khaled Eye Specialist Hospital, Riyadh, Saudi Arabia.

**Keywords:** corneal dystrophy, gelatinous drop-like corneal dystrophy, penetrating keratoplasty, subepithelial amyloid deposits, tumor-associated calcium signal transducer 2

## Abstract

**Rationale:**

Gelatinous drop-like corneal dystrophy (GDLD) is a rare autosomal recessive disorder characterized by subepithelial amyloid deposition, progressive corneal opacification, and recurrent erosions. GDLD may present during the first decade of life and interfere with visual development. It may recur after keratoplasty, complicating surgical management.

**Patient concerns:**

A 21-year-old woman of Yemeni ancestry, living in a remote area with limited access to corneal transplantation, presented with progressive left eye visual decline and foreign body sensation since early childhood. She had long-standing bilateral visual impairment and a positive family history. Her first surgery was right eye penetrating keratoplasty (PK), performed elsewhere approximately 1 year earlier for an undiagnosed opacity.

**Diagnoses:**

Examination revealed nystagmus, a clear right graft with yellowish recipient deposits, and dense limbus-to-limbus left corneal opacification with stromal haze, mulberry-like deposits, and neovascularization. Uncorrected visual acuity (VA) was 20/200 in the right eye and counting fingers in the left. Best spectacle-corrected VA was 20/100 in the right eye, while reliable refraction could not be obtained in the left. Histopathology demonstrated Congo red-positive subepithelial amyloid deposits with birefringence under polarized light, confirming GDLD. Genetic testing for tumor-associated calcium signal transducer 2 was not performed.

**Interventions:**

The left eye underwent superficial keratectomy and attempted lamellar dissection assisted by intraoperative optical coherence tomography; dense deep stromal opacity necessitated conversion to full-thickness PK.

**Outcomes:**

Both grafts remained clear without rejection during follow-up. Minimal focal subepithelial haze in a temporal area of the left donor graft was noted at the 2 most recent visits, remained stationary between them, and was clinically insignificant. At final follow-up, approximately 2 years after right eye PK and 10 months after left eye PK, best spectacle-corrected VA was 20/100 in the right eye and 20/80 − 1 in the left. Intraocular pressure was normal, and posterior pole examinations were unremarkable.

**Lessons:**

Advanced bilateral GDLD poses clinical and surgical challenges. PK may restore corneal clarity and provide meaningful visual rehabilitation, although recovery may remain incomplete after early-onset visual impairment and delayed treatment. Long-term monitoring is essential because recurrence and repeat intervention may be required. Early recognition, timely referral, and individualized management matter where donor corneal tissue access is limited.

## 1. Introduction

Corneal dystrophies, which are a heterogeneous group of primarily hereditary disorders, are characterized by progressive corneal opacification, leading to visual impairment. Gelatinous drop-like corneal dystrophy (GDLD) is a form of localized primary corneal amyloidosis, a rare but severe form of corneal dystrophy. The pathogenesis of GDLD involves the deposition of amyloid material within the corneal epithelium and stroma, leading to structural and functional impairments.^[1]^ This progressive condition typically manifests in the first 2 decades of life and is marked by recurrent corneal erosions, photophobia, significant visual impairment, and bilateral corneal opacification.^[1,2]^ The disease is associated with mutations in the tumor-associated calcium signal transducer 2 (TACSTD2) gene, which plays a critical role in epithelial integrity and function. The diagnosis is primarily based on clinical presentation, family history, and histopathological confirmation of amyloid deposits.^[2]^

Clinically, GDLD presents in different subtypes: the mulberry or nodular type, which is the most common form and manifests in early childhood with multiple elevated gelatinous deposits resembling mulberries; the band keratopathy-like type, which appears as a subepithelial corneal opacity resembling band-shaped keratopathy; the kumquat-like type, which is characterized by multiple small yellowish deposits scattered across the cornea; and the diffuse type, which is characterized by a hazy, ground-glass-like corneal opacity without distinct nodules.^[3]^

The management of GDLD remains challenging because of its progressive course, the risk of recurrence after surgical intervention, and the potential need for repeated procedures.^[3]^ In this case report, we describe a young woman with advanced bilateral GDLD, long-standing visual impairment, and limited access to corneal transplantation who underwent sequential penetrating keratoplasty (PK). The report highlights the clinical and histopathological basis of diagnosis, the intraoperative conversion from attempted lamellar dissection to PK in the left eye, and the visual outcomes after restoration of corneal clarity. It also emphasizes the importance of early recognition, timely referral, individualized surgical planning, and long-term postoperative monitoring in patients with GDLD.

## 2. Case presentation

A 21-year-old otherwise healthy woman of Yemeni ancestry, who lived in a remote area with limited access to corneal transplantation, presented with progressive bilateral visual decline, foreign body sensation and recurrent corneal erosions since early childhood. Her family also reported abnormal involuntary eye movements beginning during childhood. She had not undergone any previous ocular intervention until undergoing PK in the right eye at another facility approximately 1 year before presentation for an undiagnosed corneal opacity.

Examination revealed nystagmus. In the right eye, uncorrected visual acuity (UCVA) was 20/200, and best spectacle-corrected VA (BSCVA) was 20/100 with a manifest refraction of − 2.00 − 5.00 × 120. The right cornea showed a clear full-thickness PK graft with intact sutures and yellowish deposits at the recipient cornea. Intraocular pressure (IOP) was within normal limits, and fundus examination revealed an unremarkable posterior pole exam.

In the left eye, UCVA was counting fingers, with no improvement using pinhole testing. The cornea showed numerous whitish-yellowish nodules scattered across its surface, consistent with mulberry-like or gelatinous deposits. Diffuse stromal haze resulted in dense white limbus-to-limbus opacification, accompanied by corneal neovascularization involving all quadrants and extending centrally (Fig. 1A). The opacity precluded reliable refraction and direct visualization beyond the cornea. The potential acuity meter result was at least 20/200, and ultrasonography showed an unremarkable posterior segment and optic nerve.

**Figure 1.**
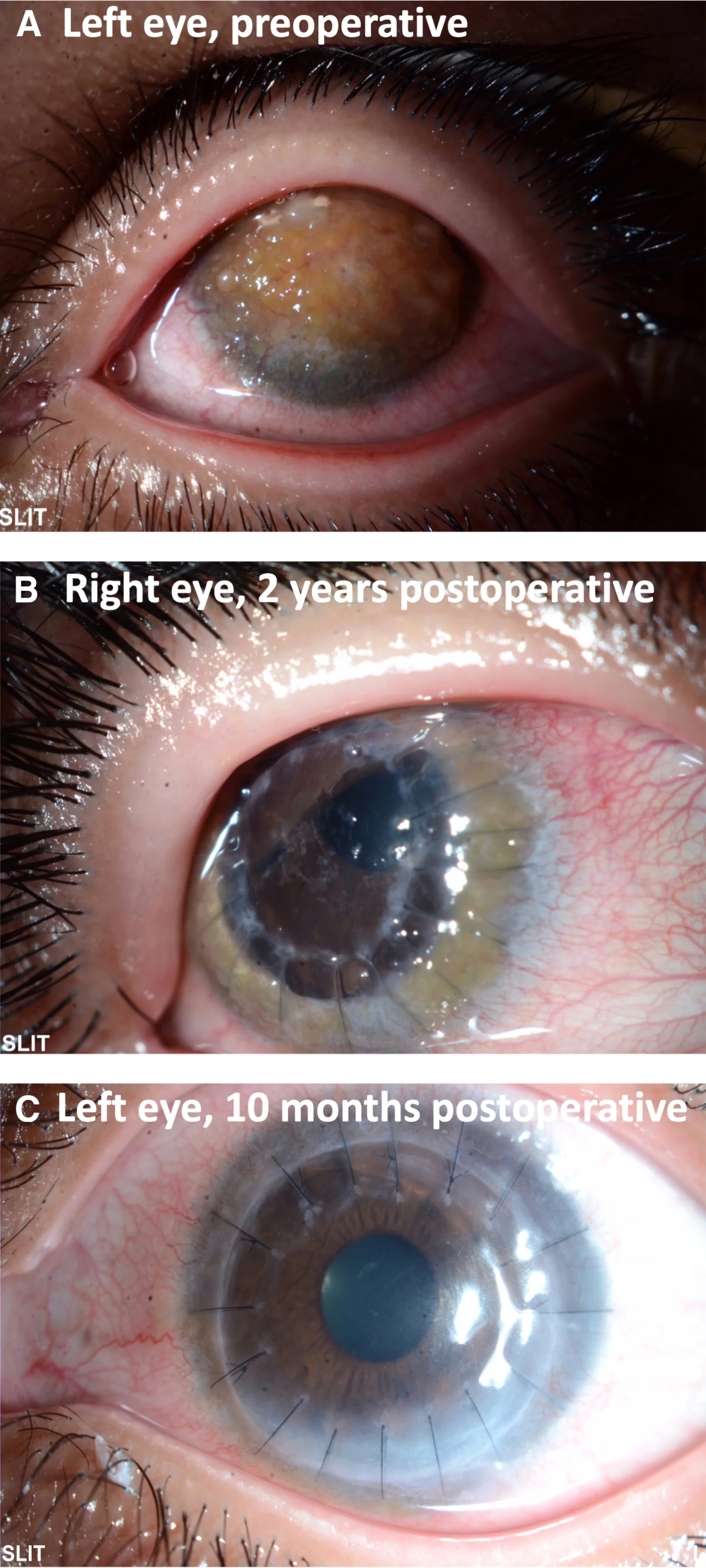
Clinical photographs of GDLD. (A) Preoperative diffuse slit-lamp photograph of the left eye showing advanced corneal opacification; (B) Diffuse slit-lamp photograph of the right eye 2 years after PK; (C) Diffuse slit-lamp photograph of the left eye 10 months after PK. GDLD = gelatinous drop-like corneal dystrophy, PK = penetrating keratoplasty.

The family history was significant for parental consanguinity. The parents are first cousins. Of 6 siblings (2 female, 4 male), both females, the patient and her sister, were reportedly affected, while the 4 males were unaffected; the patient’s maternal uncle was also reportedly affected. Only the patient and her father were examined at our institution; the father was clinically unaffected, and the remaining family history was obtained by report and not independently confirmed. Genetic counseling on inheritance and recurrence risk was provided, and TACSTD2 testing was recommended.

The combination of early-onset bilateral disease, positive family history, progressive visual impairment, and characteristic clinical findings suggested advanced GDLD. Given the recurrent corneal erosions, severe corneal opacity, visual impairment in the left eye, and limited access to corneal transplantation in the patient’s hometown, the decision was made to proceed with lamellar keratoplasty (LK), with possible intraoperative conversion to PK, under general anesthesia.

### 2.1. Surgical technique

Under sterile conditions, superficial keratectomy (SK) was performed using blunt and sharp dissection to remove the superficial corneal deposits (Figs. 2A and 2B). Bleeding from corneal neovascularization was controlled using cellulose sponges soaked in diluted epinephrine (1:10000) (Fig. 2C). Intraoperative optical coherence tomography was used to assess corneal thickness and assist in planning trephination depth (Fig. 2D). After marking the geometric center of the cornea, the recipient cornea was trephined to 70% depth using a 7.75-mm vacuum trephine (Fig. 2E).

**Figure 2.**
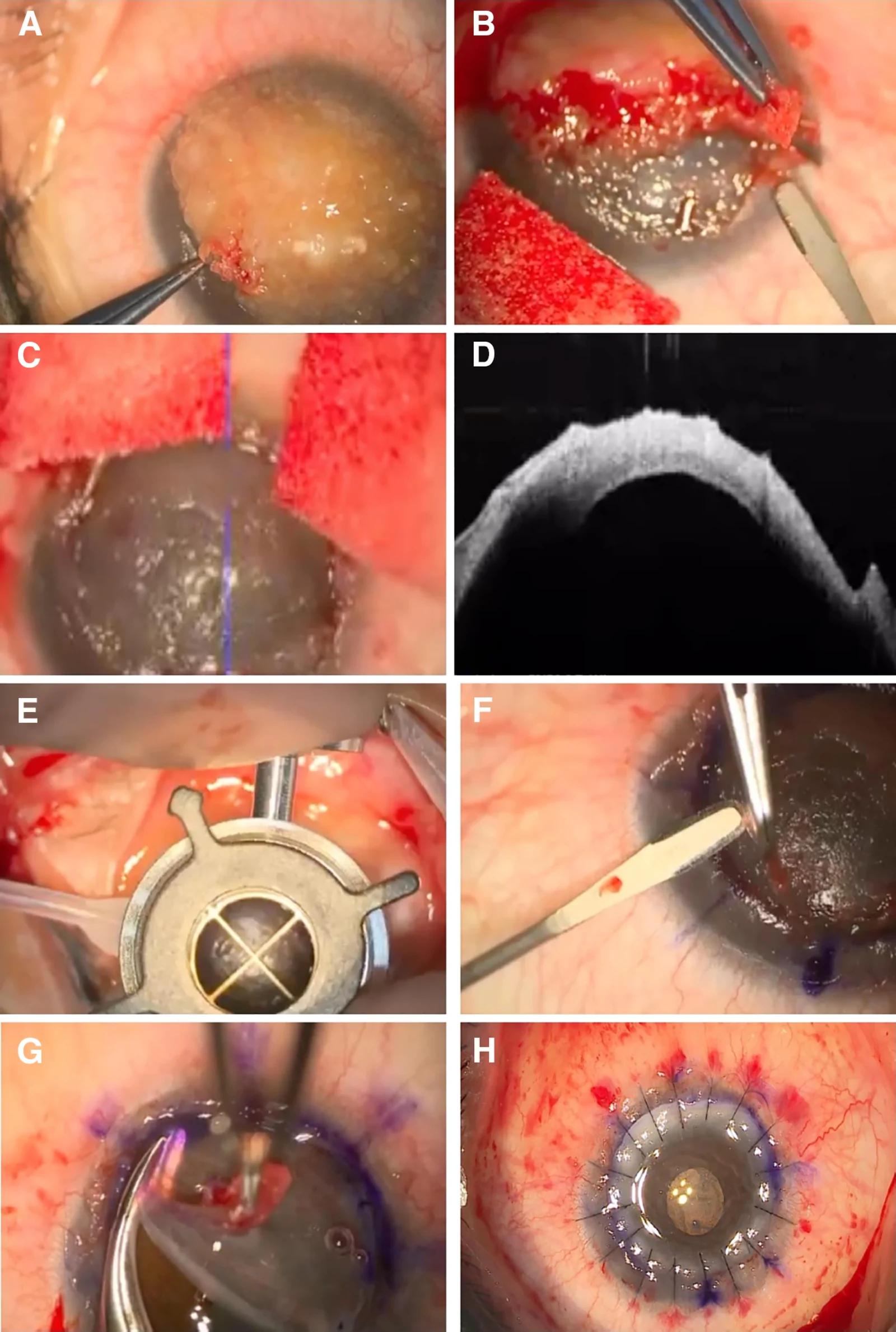
Surgical steps of PK in the left eye. (A) Identification of the surgical plane during SK; (B) lamellar dissection of the corneal opacity; (C) visualization of the underlying opaque corneal stroma; (D) iOCT image of the cornea corresponding to the level shown in panel C; (E) partial-thickness recipient corneal trephination; (F) attempted lamellar dissection through the remaining opaque stroma; (G) conversion to full-thickness PK because complete removal of the remaining opaque stroma could not be achieved by lamellar dissection; (H) placement of a full-thickness donor graft secured with 10-0 nylon sutures. iOCT = Intraoperative optical coherence tomography, PK = penetrating keratoplasty, SK = superficial keratectomy.

Manual sharp lamellar dissection was then attempted using a mini-crescent blade to remove the remaining opaque stroma (Fig. 2F). However, the deep stromal layers were densely opaque and not amenable to complete lamellar dissection. The procedure was therefore converted to full-thickness PK to avoid leaving visually significant opaque stroma (Fig. 2G).

The donor corneal tissue was obtained from an accredited eye bank and released for transplantation after standard donor screening, serological testing, and slit-lamp and specular microscopic evaluation. The donor tissue had an endothelial cell density of 2522 cells/mm^2^ and a clear zone of 9.5 mm. The donor cornea was trephined to 8.25 mm and secured in place using 16 interrupted 10-0 nylon sutures with buried knots (Fig. 2H; Video S1). The excised corneal tissue was placed in formalin and sent for histopathological examination.

**Video 1.** 

No preoperative treatment or intraoperative cauterization of the corneal neovascularization was performed. Most vessels appeared superficial and were associated with the deposits removed during SK. Treatment of any persistent neovascularization was reserved for a later stage, if required, after postoperative inflammation had subsided.

Postoperatively, the patient was monitored daily during the first 5 days of admission. UCVA in the operated left eye was 20/200, IOP was 14 mm Hg, and mild graft edema was present. Moxifloxacin eye drops were administered 4 times daily until complete epithelialization. Prednisolone acetate eye drops were prescribed every 2 hours while awake during the first postoperative week, followed by a slow taper to 4, 3, and 2 times daily, each for approximately 3 months, and subsequently once daily. The topical steroid regimen was adjusted according to the clinical findings.

### 2.2. Histopathology

The excised corneal tissue revealed an irregular anterior surface, absent Bowman layer, and disorganized overlying epithelium. Homogeneous subepithelial deposits stained positively with Congo red and exhibited birefringence under polarized light, consistent with amyloid deposition.

Based on the patient’s early-onset bilateral disease, positive family history, characteristic slit-lamp findings, and Congo red-positive subepithelial amyloid deposition, the diagnosis of GDLD was established. Molecular confirmation by TACSTD2 genetic testing was not performed.

### 2.3. Postoperative follow-up

The patient’s clinical course and postoperative follow-up are summarized in Table 1.

**Table 1:** Timeline of the patient’s clinical course, interventions, and outcomes.

Time point	Clinical event/ intervention	Key findings/ outcome
Early childhood	Onset of bilateral progressive visual impairment and foreign body sensation; abnormal involuntary eye movements developed.	No ocular intervention was performed.
1 year before presentation	Right eye PK was performed at another facility for an undiagnosed corneal opacity.	Right graft remained clear.
Presentation at age 21 years	Evaluated for advanced left eye corneal disease.	Nystagmus and positive family history. Right eye UCVA: 20/200; BSCVA: 20/100. Left eye UCVA was CF, with no improvement on pinhole testing. An attempted manifest refraction was unsuccessful because the dense corneal opacity precluded reliable measurement. Mulberry-like deposits, dense corneal opacity, and corneal neovascularization were suggestive of GDLD.
Left eye surgery	Superficial keratectomy was performed, and attempted lamellar dissection was converted to full-thickness PK.	Histopathological examination supported the diagnosis of GDLD.
First postoperative week	Daily postoperative monitoring; topical antibiotic and intensive topical steroid therapy initiated.	Left eye UCVA improved to 20/200; mild graft edema was present and IOP of 14 mm Hg.
1-month follow-up	Topical steroid QID and lubricants continued.	Both grafts were clear; left graft was fully epithelialized. IOP was within normal limits, and fundus examination was unremarkable bilaterally. UCVA was 20/200 in both eyes; left eye refraction was not performed.
3-month follow-up	Topical steroid reduced to TID.	At the 3-month follow-up visit, UCVA was 20/200 in both eyes and BSCVA was 20/100 in the right eye and 20/80 − 1 in the left eye. Manifest refraction was − 2.00 − 5.00 × 120 in the right eye and − 3.00 − 8.00 × 070 in the left eye. Automated keratometry showed K1 42.00 D and K2 48.00 D in the right eye, with 6.00 D of corneal cylinder at 015°, and K1 40.00 D and K2 49.00 D in the left eye, with 9.00 D of corneal cylinder at 140°.
Last follow-up 2 years after right eye PK and 10 months after left eye PK	Topical steroid reduced to QD; no additional topical immunomodulators were initiated.	UCVA was 20/200 in both eyes. BSCVA was clinically unchanged at 20/100 in the right eye and 20/80 − 1 in the left eye. Manifest refraction was − 1.75 − 4.50 × 115 in the right eye and − 3.50 − 7.75 × 085 in the left eye. An RGP contact lens trial with over-refraction provided no additional improvement. Both grafts remained clear except for minimal focal subepithelial haze in a temporal area of the left donor graft, noted at the last 2 visits and stationary between them, judged clinically insignificant. IOP was within normal limits, and no rejection episodes occurred.

K1/K2 represents flattest/steepest keratometry.

BSCVA = best spectacle-corrected visual acuity, CF = counting fingers, D = diopters, GDLD = gelatinous drop-like corneal dystrophy, IOP = intraocular pressure, PK = penetrating keratoplasty, QD = once daily, QID = four times daily, RGP = rigid gas-permeable, TID = three times daily, UCVA = uncorrected visual acuity.

At the 1-month follow-up visit, the patient reported no complaints. UCVA was 20/200 in both eyes. BSCVA in the right eye remained stable at 20/100, similar to presentation, while refraction was not performed for the left eye at this visit. The right graft was clear with intact sutures. The left graft was also clear with intact sutures and complete epithelialization. IOP was within normal limits, and fundus examination was unremarkable in both eyes. The patient was maintained on topical steroid therapy 4 times daily and lubricating eye drops.

At the 3-month follow-up visit, UCVA was 20/200 in both eyes. BSCVA was 20/100 in the right eye and 20/80 − 1 in the left eye. Manifest refraction was − 2.00 − 5.00 × 120 in the right eye and − 3.00 − 8.00 × 070 in the left eye. Automated keratometry of the right eye showed K1 of 42.00 D and K2 of 48.00 D, with 6.00 D of corneal cylinder at 015°. Automated keratometry of the left eye showed K1 of 40.00 D and K2 of 49.00 D, with 9.00 D of corneal cylinder at 140°. Surgically induced astigmatism could not be calculated because reliable preoperative keratometry or corneal topography was unavailable for comparison. Reliable corneal topography/tomography and graft pachymetry could not be acquired because nystagmus and limited fixation affected image acquisition; however, automated keratometry readings were obtainable. Keratometry was obtained using a Topcon KR-800 autorefractor-keratometer (Topcon Corporation) and, to accommodate her nystagmus and head positioning, a handheld Nidek Handy Ref-K autorefractor/keratometer (Nidek Co., Ltd.). To minimize motion artifact, both eyes were kept open with a visible fixation target, and multiple readings were acquired during foveation periods; a trial of fogging the fellow eye was also used to slightly dampen the nystagmus during acquisition. Readings with optimal mire quality and consistency were selected to obtain reproducible keratometry values. Topical steroid therapy was reduced to 3 times daily, and lubricating eye drops were continued.

The patient was subsequently followed every 3 months. At the last follow-up, approximately 2 years after right eye PK and 10 months after left eye PK, UCVA was 20/200 in both eyes. BSCVA remained stable at 20/100 in the right eye and 20/80 − 1 in the left eye, with clinically unchanged manifest refraction. Manifest refraction was − 1.75 − 4.50 × 115 in the right eye and − 3.50 − 7.75 × 085 in the left eye. A rigid gas-permeable trial contact lens over-refraction was performed but yielded no additional visual improvement beyond the measured BSCVA. Both grafts remained clear, except for minimal focal subepithelial haze in a temporal area of the left donor graft. This haze remained stationary between the last 2 visits, was not associated with the gelatinous deposits characteristic of amyloid, and did not affect best-corrected VA; it was therefore considered clinically insignificant, and continued surveillance was planned. All sutures remained in place, and IOP was within normal limits (Figs. 1B and 1C). The patient had reached a once-daily topical steroid regimen. Complete suture removal from both eyes under anesthesia was planned because significant nystagmus made slit-lamp suture removal difficult.

During follow-up, medication adherence and tolerability were assessed by patient report. The patient reported good adherence to the prescribed regimen, including the steroid taper, and no drop-related intolerance. No adverse or unanticipated events occurred during the follow-up period.

Given the higher-risk graft, the patient was maintained on close follow-up every 3 months with counseling on the warning symptoms of graft rejection and was given access to the ophthalmic emergency service for prompt evaluation and treatment should early complications or rejection arise.

## 3. Discussion

GDLD is an autosomal recessive subepithelial corneal dystrophy characterized by progressive amyloid deposition within the cornea.^[4]^ Its prevalence is higher in Japan than in other regions.^[5]^ This increased prevalence has primarily been attributed to a founder effect and regional genetic clustering rather than consanguinity alone. Most affected Japanese patients have been reported to share the same haplotype associated with the Q118X mutation in TACSTD2.^[6]^ In regions where consanguineous marriage is common, autosomal recessive disorders may be encountered more frequently, and familial clustering should prompt consideration of inherited corneal dystrophies.

The Arabian Peninsula, including Saudi Arabia and Yemen, has a high prevalence of consanguineous marriage, which is associated with an increased occurrence of autosomal recessive disorders.^[7]^ Although the prevalence and genetic characteristics of GDLD in the region remain poorly documented, consanguinity may contribute to the occurrence and familial clustering of this autosomal recessive condition. The reported affection pattern is consistent with autosomal recessive inheritance in a consanguineous family. This further emphasizes the importance of obtaining a detailed family history and considering genetic counseling and molecular testing in suspected familial cases.

Clinical manifestations of GDLD commonly begin during childhood, often before 10 years of age, and include photophobia, epiphora, foreign body sensation, recurrent corneal erosions, and progressive visual impairment.^[8]^ Because disease onset may occur during the period of visual development, early recognition and continued ophthalmic care are particularly important. In the present case, visual impairment began in early childhood, but the patient received no ocular surgical intervention until right eye PK at 20 years of age, approximately 1 year before presentation. The presence of nystagmus, severe bilateral corneal opacity, and incomplete visual recovery despite restoration of corneal clarity raised concern for impaired visual development. However, the precise contribution of delayed intervention to the final visual outcome cannot be established from a single case. The presence of a similarly affected sibling further supports a hereditary etiology and emphasizes the importance of early ophthalmic assessment in patients with a positive family history.

The differential diagnosis included other amyloid-associated corneal disorders, particularly lattice corneal dystrophy types I and IIIA and secondary corneal amyloidosis. Lattice corneal dystrophy was considered less likely because the patient lacked characteristic branching stromal lattice lines and instead demonstrated diffuse mulberry-like subepithelial gelatinous deposits.^[4]^ Furthermore, unlike GDLD, lattice corneal dystrophy has an autosomal dominant inheritance pattern.^[4]^ Secondary corneal amyloidosis was also considered unlikely because of the bilateral early-onset presentation, positive family history, and absence of preceding chronic inflammatory, infectious, traumatic, or degenerative ocular disease.^[9]^ Küchlin et al described the distinct histopathological characteristics of amyloid deposits in GDLD compared with other amyloid-associated corneal dystrophies, including lattice and Avellino dystrophies.^[10]^ In GDLD, amyloid deposits are typically extensive and concentrated within the subepithelial region, whereas lattice corneal dystrophy is characterized by more dispersed stromal amyloid deposition in a branching or patchy distribution.^[10]^ In our patient, histopathological examination demonstrated homogeneous subepithelial Congo red-positive amyloid deposits with birefringence under polarized light. Together with the early-onset bilateral disease, positive family history, and characteristic slit-lamp appearance, these findings strongly supported the diagnosis of GDLD despite the absence of molecular confirmation.

Management of GDLD is challenging because of its progressive and recurrent nature and the potential need for repeated interventions to preserve vision and quality of life.^[10–13]^ Conservative approaches, including artificial tears, soft bandage contact lenses, and SK, may be used during earlier stages or following recurrence to delay major surgery and prolong the surgery-free interval.^[10,14]^ A retrospective study of 20 patients with GDLD involving 40 eyes demonstrated slower progression of corneal damage and a significantly prolonged surgery-free interval in eyes treated with soft contact lenses.^[14]^ In advanced disease, surgical options include LK and PK, with the choice influenced by the depth and extent of stromal involvement. Boston type I keratoprosthesis (KPro) has also been reported as an alternative option in selected cases.^[15]^

The timing of intervention is important and must be balanced against the risk of recurrence and the potential need for repeated surgery. Although direct comparison between Boston type I KPro and PK is limited by substantial differences in indication, mechanism, and postoperative care requirements, the report by Cortina et al highlights the importance of early visual rehabilitation in GDLD.^[15]^ Their patient achieved a VA of 20/20 at 1 month after Boston type I KPro implantation and had undergone multiple superficial keratectomies beginning at 8 years of age.^[15]^ In contrast, our patient presented in early adulthood with advanced bilateral disease and had received no ocular intervention until shortly before presentation. Although VA improved substantially following sequential PK, further improvement could not be achieved even with contact lens fitting. The final visual outcome may have been influenced by several factors, including the timing of intervention and the duration of visual impairment before treatment.

Similarly, Samira et al reported 3 male siblings with severe GDLD who underwent full-thickness PK after presenting with marked corneal deposits and visual impairment without previous intervention.^[8]^ All 3 siblings presented with baseline VA limited to light perception with good projection. One month following PK, VA improved in all 3 patients to 3/60, 2/60, and 2/60, respectively. These findings, together with the present case, suggest that restoration of corneal clarity may support meaningful visual rehabilitation, while final visual recovery may remain incomplete in eyes with early-onset visual impairment and delayed access to treatment.

Shimazaki et al retrospectively reviewed 7 patients with GDLD who underwent 35 keratoplasties, including 22 PK procedures and 13 LK procedures, during prolonged follow-up.^[16]^ Severe recurrence occurred following each keratoplasty. They reported 11 graft rejection episodes, most of which involved endothelial rejection, and endothelial decompensation in 2 grafts. Compared with PK, LK offered the advantage of avoiding endothelial rejection, which occurred in 8 of the 22 PK grafts (36.4%).^[16]^ Although PK can initially restore corneal clarity and improve vision, recurrence of amyloid deposition is common because the host epithelium eventually repopulates the donor graft. Subepithelial haze may recur within the first postoperative year, with more substantial amyloid accumulation reported at approximately 26 months.^[16]^ In our patient, the minimal focal subepithelial haze in a temporal area of the donor graft remained stationary over the 2 most recent visits, was not associated with the gelatinous morphology of amyloid deposits, and did not affect VA. While these features favor a nonspecific, clinically insignificant change rather than early amyloid recurrence, the recurrent nature of GDLD warrants continued long-term surveillance, and recurrence cannot be excluded on the basis of the current follow-up.

No rejection episodes were encountered, and topical steroid therapy was gradually tapered to once daily without the use of additional topical immunomodulators. These findings represent short- to intermediate-term anatomical and visual outcomes. Given the preoperative corneal vascularization, this graft was considered high-risk for rejection. A topical calcineurin inhibitor (cyclosporine or tacrolimus) may therefore be considered during subsequent follow-up as an additional steroid-sparing option if clinically indicated. Residual corneal neovascularization can be monitored and managed with medical or surgical measures as needed.^[17,18]^

Sequential bilateral PK is an established surgical approach for advanced GDLD and is not presented as a novel technique in this report. The clinical value of this case lies in documenting advanced bilateral disease in a young adult with early-onset visual impairment, delayed access to corneal transplantation, nystagmus, detailed intraoperative decision-making, histopathological confirmation, and bilateral postoperative follow-up. The case also demonstrates that attempted lamellar dissection may require intraoperative conversion to full-thickness PK when deep stromal opacity prevents adequate removal of diseased tissue.

Several limitations should be acknowledged. Molecular confirmation with TACSTD2 genetic testing was not performed. Molecular analysis was recommended but was initially unavailable because of resource limitations, and the patient and family subsequently elected not to proceed with testing. Nevertheless, the combination of early-onset bilateral disease, positive family history, characteristic slit-lamp findings, and Congo red-positive subepithelial amyloid deposition strongly supported the diagnosis of GDLD. Although automated keratometry readings were obtained, reliable corneal topography/tomography and graft pachymetry could not be acquired because significant nystagmus and limited fixation affected image acquisition. Furthermore, the relatively short follow-up duration, particularly for the left eye, limits conclusions regarding long-term graft survival and recurrence. Continued longitudinal monitoring is therefore necessary.

## 4. Conclusion

Keratoplasty can restore corneal clarity and provide meaningful visual rehabilitation in advanced GDLD; however, final visual recovery may remain incomplete in eyes with early-onset visual impairment and delayed access to treatment. Advanced GDLD requires individualized surgical planning because the depth and extent of corneal involvement may not be fully apparent preoperatively. Intraoperative optical coherence tomography aided in assessing residual stromal opacity and corneal thickness during surgery, informing the intraoperative decision to convert from attempted lamellar dissection to full-thickness PK when complete removal of the affected stroma could not be achieved by lamellar dissection. Because recurrence may occur after keratoplasty, management should balance timely intervention with the potential need for repeated procedures and prolonged postoperative surveillance. Early recognition and referral are particularly important in settings where access to donor corneal tissue is limited.

## Author contributions

**Conceptualization:** Mohammed M. Abusayf, Majed AlKharashi.

**Data curation:** Rana Alhumaemydi, Abeer Alkhodier.

**Investigation:** Rana Alhumaemydi, Abeer Alkhodier, Majed AlKharashi.

**Methodology:** Mohammed M. Abusayf.

**Project administration:** Mohammed M. Abusayf.

**Supervision:** Mohammed M. Abusayf, Majed AlKharashi.

**Writing – review & editing:** Mohammed M. Abusayf, Rana Alhumaemydi.

**Writing – original draft:** Rana Alhumaemydi, Abeer Alkhodier.
